# Prevalence of internet addiction and associated factors among university students in Ethiopia: systematic review and meta-analysis

**DOI:** 10.3389/fdgth.2024.1373735

**Published:** 2024-09-11

**Authors:** Yibeltal Assefa Atalay

**Affiliations:** School of Public Health, College of Health Science and Medicine, Wolaita Sodo University, Wolaita Sodo, Ethiopia

**Keywords:** prevalence, internet addiction, factors, students, systematic review, meta-analysis, Ethiopia

## Abstract

**Introduction:**

Internet addiction refers to the excessive and uncontrolled utilization of the Internet, which disrupts one's daily activities. The current state of knowledge regarding internet addiction in Ethiopia is limited. Consequently, the objective of this study is to ascertain the combined prevalence of Internet addiction and its correlated factors among university students in Ethiopia.

**Methods:**

To identify potential research findings, an extensive literature search was conducted using electronic databases such as PubMed/MEDLINE, Web of Science, and Google Scholar. The presence of heterogeneity between studies was assessed using Cochrane Q test statistics and I2 test statistics, while the effects of small studies were examined using Eggers statistical tests at a 5% significance level. Additionally, a sensitivity analysis was carried out. A random effects model was used to estimate the pooled prevalence and associated factors of Internet addiction among students. The primary focus of this research was to determine the prevalence of Internet addiction, while the secondary aim was to identify the factors associated with Internet addiction.

**Results:**

To determine the overall prevalence of Internet addiction among university students in Ethiopia, a comprehensive analysis of 11 studies was conducted. The results of this study show that the pooled prevalence of Internet addiction was 43.42% (95% CI: 28.54, 58.31). The results also suggest that certain factors such as online gaming, depression, and current khat chewing are significantly associated with internet addiction among university students.

**Conclusions:**

In Ethiopia, about one-third of university students suffer from internet addiction. The prevalence of Internet addiction among Ethiopian students is associated with online gaming, depression, and concurrent khat consumption. Therefore, we strongly recommend that health planners and policymakers prioritize monitoring and addressing Internet use and addiction in the Ethiopian context.

## 1 Introduction

The ubiquitous nature of the Internet has made it an integral part of everyday human life and provides easy access to entertainment on global scale (1). The number of people using the Internet every day is over three billion worldwide, with the younger generation being the most frequent users (2). The Internet has become an indispensable tool in various fields, including evidence-based medicine, research, and learning, access to medical and online databases, and management of patients in remote locations, as well as academic and recreational activities in the health sector (2, 3). However, excessive use of the Internet has been linked to addiction and mental health problems, highlighting the negative consequences of its use (4).

Internet addiction (IA) is a multifaceted problem that impacts a range of online activities, including social networking, online gaming, and online shopping (5). Since 2013, Internet gaming disorder has been recognized as a significant clinical problem in both the American Psychiatric Association's DSM-5 classification of mental and behavioral disorders and the 11th edition of the World Health Organization's International Classification of Diseases (ICD-11) (6).

Despite their wide range of manifestations, neither the International Classification of Diseases, 11th Edition (ICD-11), nor the Diagnostic and Statistical Manual of Mental Disorders, 5th Edition (DSM-5) include Internet addiction (IA) as a clear diagnosis or Condition (7). IA is characterized by excessive and uncontrolled internet use that has a detrimental effect on a person's daily functioning. This problem is closely related to other mental disorders such as depression and attention deficit hyperactivity disorder. Global estimates suggest that IA affects approximately 6% of the population, with prevalence rates ranging from 2.6% in Western Europe to 10.25% in the Middle East (8).

Furthermore, it is worth noting that Internet addiction (IA) has emerged as a significant mental health problem among Chinese adolescents. Recent research has found that a significant proportion of Chinese college students, 10.6%–13.6%, were classified as Internet addicts (9). Similarly, a study conducted among college students in Taiwan found a prevalence rate of 15.3% for Internet addiction (10).

Problematic Internet Use (PIU) is more common among university students, as shown by studies of Malaysian medical students and Greek university students. Among Malaysian medical students, the prevalence of PIU ranged from 36.9% to 81%, with cut-offs of 43% and 31% to 79%, respectively (11, 12), as assessed by the study instruments Internet Addiction Questionnaire and Internet Addiction Diagnostic Questionnaire. Similarly, Greek university students had a PIU prevalence ranging from 12% to 34.7% as measured by the study instrument Problematic Internet Use Diagnostic Test, although no specific cut-off value was established (13). Teens are particularly more prone to developing internet addiction due to their extensive use of social media platforms such as Twitter, Facebook, and Telegram for communication and gaming purposes (14).

The prevalence of Internet addiction in developing countries represents a significant public health problem, but there is limited knowledge of its prevalence in these regions, particularly Ethiopia.

Therefore, a reliable assessment of the pooled prevalence of internet addiction among university students is needed to develop targeted interventions that address its associated factors. This assessment will also serve as a basis for the development of national and international plans, procedures, and policies. Additionally, the results of this study will have significant implications for health planners and policy makers in preventing IA among students in Ethiopia. Therefore, this research aims to determine the pooled prevalence of Internet addiction and its associated factors among university students in Ethiopia.

## 2 Methods

### 2.1 Study protocol registration and reporting

The purpose of this study was to conduct a systematic review and meta-analysis to ascertain the prevalence of Internet addiction and its associated factors among university students in Ethiopia. The standard PRISMA checklist guideline (15) (Supplementary file 1) was utilized to ensure the methodological rigor of this research.

### 2.2 Searching strategy

The present study aimed to determine the prevalence of Internet addiction and associated factors among university students in Ethiopia. To achieve this, international online databases such as Pub Med, Science Direct, Scopus, and Google Scholar were used. Additionally, gray literature was retrieved from reputed universities such as Walden University in the United States of America, and the University of Ghana online institutional research repository. The search strategy was designed using the Boolean operators “AND” and “OR” and pre-identified search terms were used to ensure a comprehensive search strategy that included all relevant studies. The pre-identified search terms were “internet addiction”, “internet addiction disorder”, “problematic internet use”, and “media addiction”. The search period extended from July 1, 2023, to August 10, 2023.

### 2.3 PECO guidelines

Population: The study will focus on university students enrolled in diverse vocational courses in Ethiopia, with no limitations on the specific types of vocational courses. Exposure/Intervention: The extent of internet excessive use among the participants will be assessed using the Young Internet Addiction Test (Y-IAT). Context: The study will be conducted within the Ethiopian educational setting, specifically targeting university students. Condition: The research will solely concentrate on university students as the primary subjects of investigation.

### 2.4 Measurement of the outcome variables

The primary objective of this review was to determine the prevalence of Internet addiction, as measured by the Young Internet Addiction Test (YIAT). The severity of Internet addiction was evaluated using the Internet Addiction Test (IAT) questionnaire, which consists of 20 items that assess symptoms of Internet addiction on a scale ranging from 0 to 5. The scoring system for the IAT questionnaire is as follows: 0 (not applicable), 1 (rarely), 2 (occasionally), 3 (often), 4 (often), and 5 (always). Based on the total score, individuals were categorized into three groups: average internet users (scores 20–49), potentially problematic internet users (scores 50–79), and severe internet addicts (scores 80–100). To ensure consistency across all included studies, standardized cutoff values were applied. Scores below 50 were classified as indicative of normal Internet use, while scores above 50 were indicative of Internet addiction (2).

The secondary objective of this review was to identify the factors associated with Internet addiction. This was determined by calculating the odds ratio based on the binary outcomes of the primary studies included in the review.

### 2.5 Inclusion and exclusion criteria

This study aimed to examine the prevalence of Internet addiction (IA) among university students in Ethiopia using the Young Internet Addiction Test (YIAT). The inclusion criteria for this review were studies that reported the prevalence of IA among university students in Ethiopia using the YIAT and full-text articles written in English. Both published and unpublished articles were included, as were studies that used probability and non-probability sampling methods. However, studies reporting on IA conducted outside of university students and epidemiological studies conducted on students from school-age adolescents without mentioning Y-IAT cutoffs or using other screening tools for IA were excluded from this review.

### 2.6 Quality assessment

Author, YAA, assessed the quality of studies using the standardized quality assessment checklist developed by the Joanna Briggs Institute (JBI) (16). The critical analysis checklist consists of eight parameters, each with the options Yes, No, Unclear, and Not applicable. These parameters address specific issues related to the study's methodology. For example, they ask whether the criteria for inclusion in the sample were clearly defined, whether the study participants and setting were described in detail, whether the measurement of exposure was valid and reliable, whether the main lenses and standard criteria were used to measure the event, whether confounding factors identified, whether strategies to address confounding factors were mentioned, whether outcomes were measured accurately and reliably, and whether statistical analysis was appropriate.

A scoring system was used to determine the risk level of the studies. Studies that scored 50% and above on the quality assessment indicators were classified as low-risk studies. These results can be found in an additional file, (Supplementary file 2).

### 2.7 Risk of bias assessment

In this study, we used the methods described by Hoy et al. to develop assessment tools (17). To assess the internal and external validity of research using a set of ten criteria to determine the potential for bias. These criteria covered various aspects, including (1) the representation of the population, (2) the sampling frame used, (3) the methods used for participant selection, (4) the possibility of non-response bias, (5) the direct collection of data from subjects, (6) the acceptability of the case definition, (7) the reliability and validity of the study instruments, (8) the type of data collection used, (9) the duration of the prevalence period and (10) the appropriateness of the numerator and denominator. Each of these elements was assessed and assigned a risk of bias rated as either low or high. Studies that lacked clear assessment tools for data collection were classified as having a high risk of bias. Finally, an overall risk of bias rating was determined based on the number of studies found to be at high risk of bias, with classifications low (2), moderate (3–4), and high (5) assigned accordingly (Supplementary file 3).

### 2.8 Data extraction

Following a pre-established data extraction format, researcher (YAA) systematically collected all necessary data. The information extracted included the first author's name or research group, publication year, geographical region of the study, study setting, study design, sample size, and the status of impact assessment. The reviewers independently gathered data on factors that were found to be associated with the prevalence of IA. For the second outcome, which focused on factors related to IA, data were extracted and organized in a 2 × 2 tabular format. Subsequently, the odds ratio for each factor was calculated based on the original study results.

### 2.9 Statistical methods and data analysis

The essential information was extracted utilizing Microsoft Excel 2010 and transferred to Stata 14 for further analysis. The prevalence of IA in each primary article was determined by the authors through the calculation of standard errors using a binomial formula. We also checked the level of Heterogeneity among the reported prevalence of the studies using the Cochrane Q^2^ and I^2^ statistics. The heterogeneity was quantified as high (considerable), moderate, low as 75% and more, 50%-75%, and 25% and less respectively. The random effects model was used to estimate the der Simonian and Laird's pooled effects since test statics showed there was considerable heterogeneity among studies. The publication bias was conducted using a subjective funnel plot and objectively using Egger's test with a 5% significant level. In Egger's test *p*-value, less than 5% indicates the presence of publication bias. In addition, subgroup analysis was done using region, study setting, and sampling methods of studies to reduce the random heterogeneity between the estimates of the primary studies.

## 3 Results

### 3.1 Searching process

**Figure 1:**
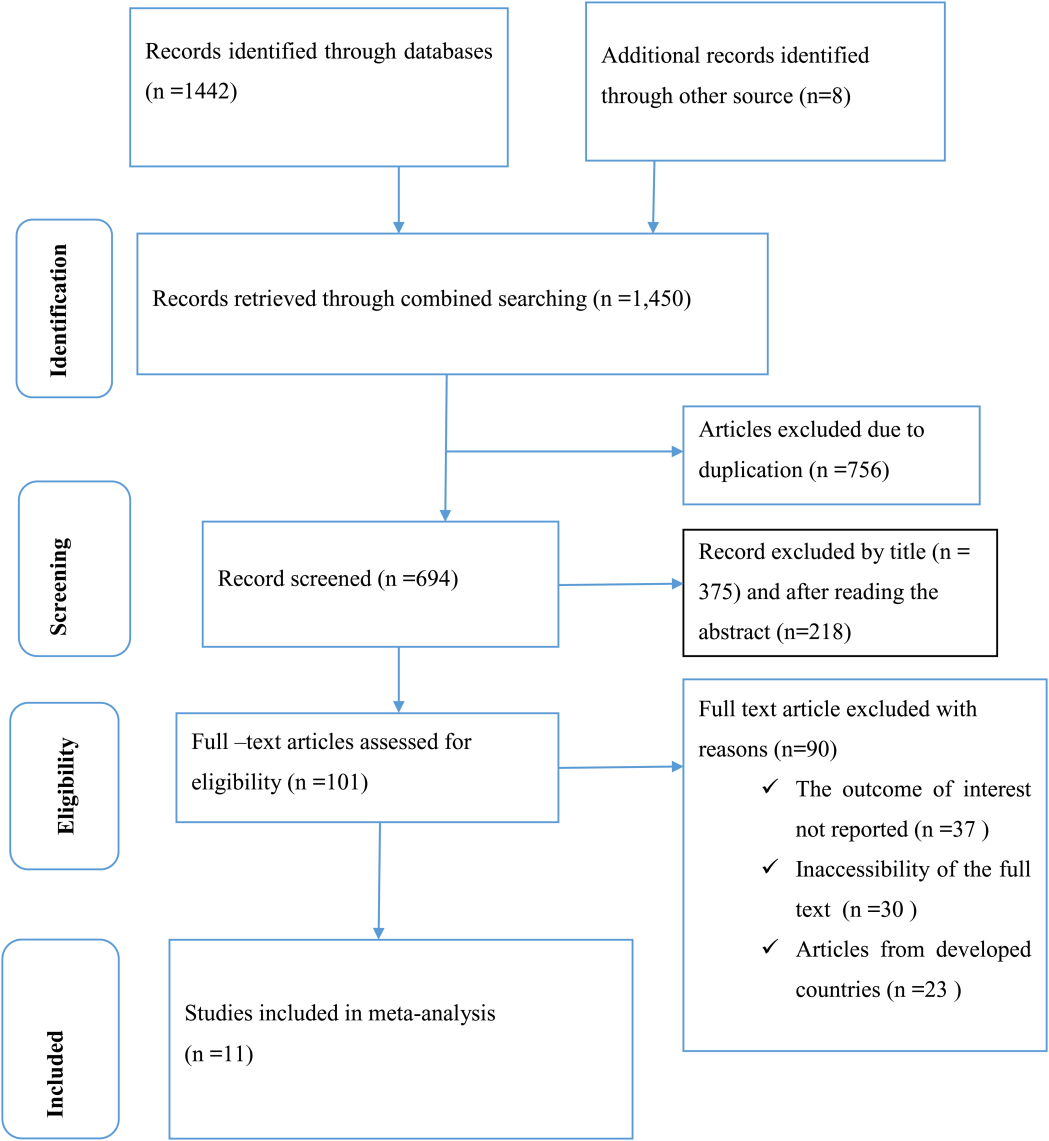
PRISMA flow chart displays the process of search and selection of studies.

A total of 8 and 1,442 articles were identified through the use of database and manual search methods. After removing duplicate studies, 694 studies were selected for screening based on their full titles and abstracts. Of these, 594 studies were excluded based on their titles and abstracts, meaning that 101 articles had to be assessed as full-text articles. After a thorough review of the full text, 90 articles were excluded due to a lack of full titles and abstracts and a lack of reporting of results from developed countries. Ultimately, 11 articles (18–28) with a total of 6,501 study participants were deemed eligible for inclusion in this systematic review and meta-analysis study, (Figure 1).

### 3.2 Characteristics of included studies

This systematic review and meta-analysis included 11 articles with a total sample size of 6,501 students (18–28). All studies included in this review were cross-sectional studies conducted in institutional settings. Of these cross-sectional studies, eight used probability sampling methods, while three used non-probability sampling methods. The earliest study was conducted in 2015 (23), and the most recent articles (22) were published in 2023. Specifically, five studies were conducted in the Oromia region (18, 21, 22, 24, 28), four studies in Amhara (19, 20, 23, 25), one study in Addis Ababa (26), and one study in Tigray (27). The sample sizes of the included studies ranged from 149 to 919. The Joanna Briggs Institute (JBI) quality assessment checklist was used to assess the quality of the studies and all studies were found to be at low risk of bias (Table 1).

**Table 1:** Description of studies measuring the prevalence of internet addiction among university students in Ethiopia.

Authors (Pub. year)	Region	Study setting	Study design	Sample size	Prevalence (%)	Sampling techniques	Study quality
Gurmu T. et al. (18)	Oromia	Institutional	CS	253	79	Probability	Low risk
Asrese K. et al. (19)	Amhara	Institutional	CS	812	46	Probability	Low risk
Nebiyu M.et al. (20)	Amhara	Institutional	CS	761	19.4	None-probability	Low risk
Behre Dari et al. (2022) (21)	Oromia	Institutional	CS	216	57.9	Probability	Low risk
Abdulkerim A. et al. (2023) (22)	Oromia	Institutional	CS	772	53.6	Probability	Low risk
Berihun A. et al. (2015) (23)	Amhara	Institutional	CS	836	40.9	Probability	Low risk
Tilahun E. et al. (2021) (24)	Oromia	Institutional	CS	149	21.3	None-probability	Low risk
Yosef Z. et al. (2021) (25)	Amhara	Institutional	CS	603	85	Probability	Low risk
Tsegay L. et al. (2020) (26)	Addis A.	Institutional	CS	423	11.4	Probability	Low risk
Adis Brhane et al. (2019) (27)	Tigray	Institutional	CS	919	39.6	Non-probability	Low risk
Henock A. et al. (2020) (28)	Oromia	Institutional	CS	757	23.7	Probability	Low risk

CS, cross-sectional.

### 3.3 Prevalence of IA among university students in Ethiopia

**Figure 2:**
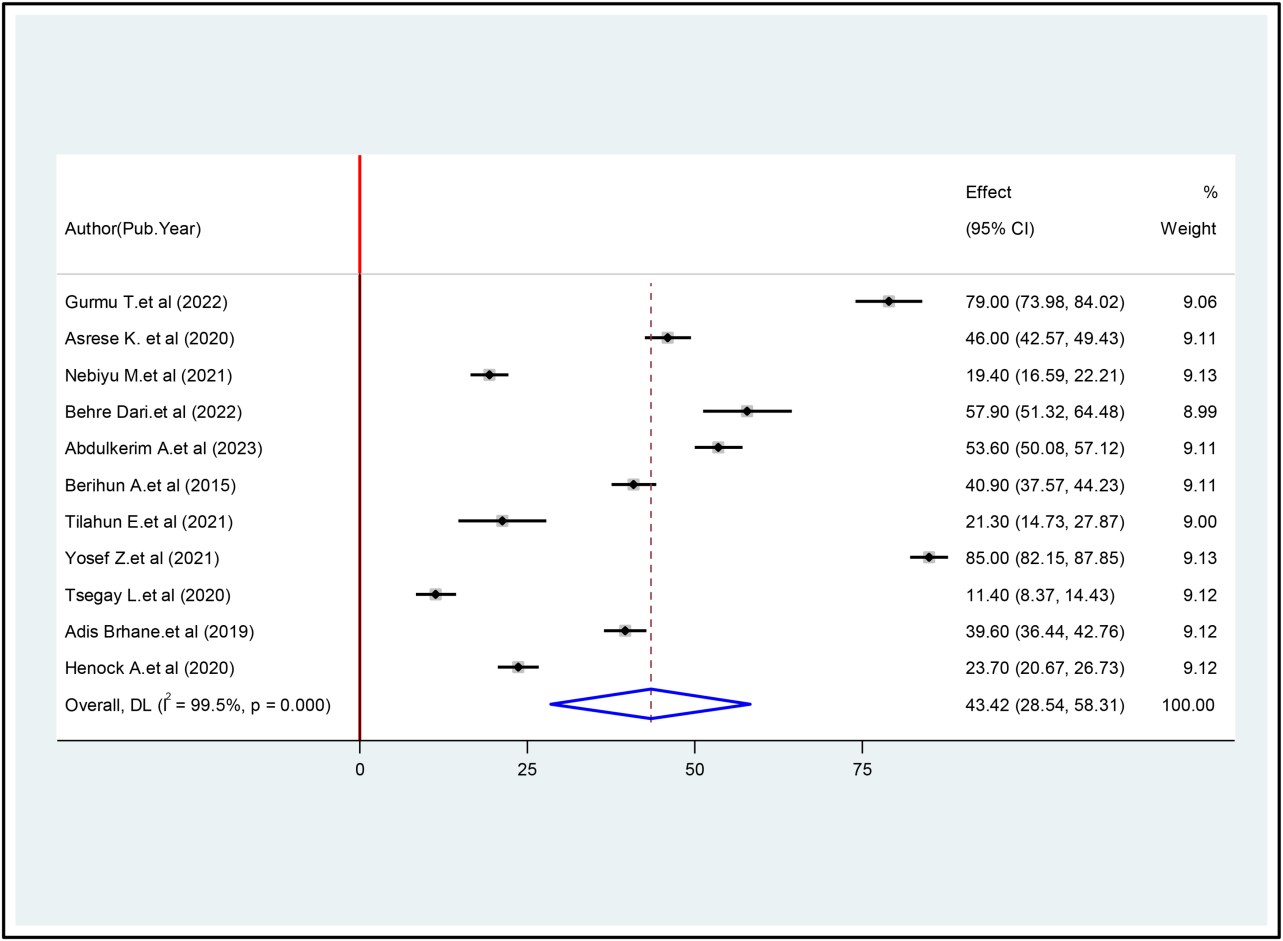
The prevalence of internet addiction among university students in Ethiopia.

The present study used a fixed-effects inverse variance model to estimate the pooled prevalence of Internet addiction (IA) among university students. This approach was considered necessary due to the high heterogeneity between studies (I^2^ = 99.5%, *P* < 0.001). A random effects model was then used to determine the overall pooled prevalence of IA among students in Ethiopia, which was 43.42% (95% CI: 28.54, 58.31) (Figure 2).

### 3.4 Subgroup analysis

A subgroup analysis was conducted to examine the impact of region and sampling methods on the prevalence of Internet addiction. In terms of region, the study conducted in Amhara revealed the highest prevalence of Internet addiction at 47.83% (95% CI = 18.78, 76.87), while the lowest prevalence was observed in Addis A. at 11.40% (95% CI = 8.37, 14.43). Furthermore, a subgroup analysis was performed based on the sampling method employed. The prevalence of Internet addiction among studies that utilized probability sampling was relatively higher at 49.66% (95% CI = 30.62, 68.69) compared to those that employed non-probability sampling, which had a prevalence of 26.85% (95% CI = 12.19, 41.51) (Table 2).

**Table 2:** Subgroup analysis of internet addiction among university students in Ethiopia (*n* = 11).

Variables	Characteristics	Included studies	Sample size	Prevalence with (%) (95% CI)	Weights
Region	Oromia	5	2,147	47.09 (25.81, 68.37)	45.28
	Amhara	4	3,012	47.83 (18.78, 76.87)	36.48
	Addis A.	1	423	11.40 (8.37, 14.43)	9.12
	Tigray	1	919	39.60 (36.44, 42.76)	9.12
Sampling techniques	Probability sampling	8	4,672	49.66 (30.62, 68.69)	72.76
	Non-probability sampling	3	1,829	26.85 (12.19, 41.51)	27.24

### 3.5 Sensitivity analysis

**Figure 3:**
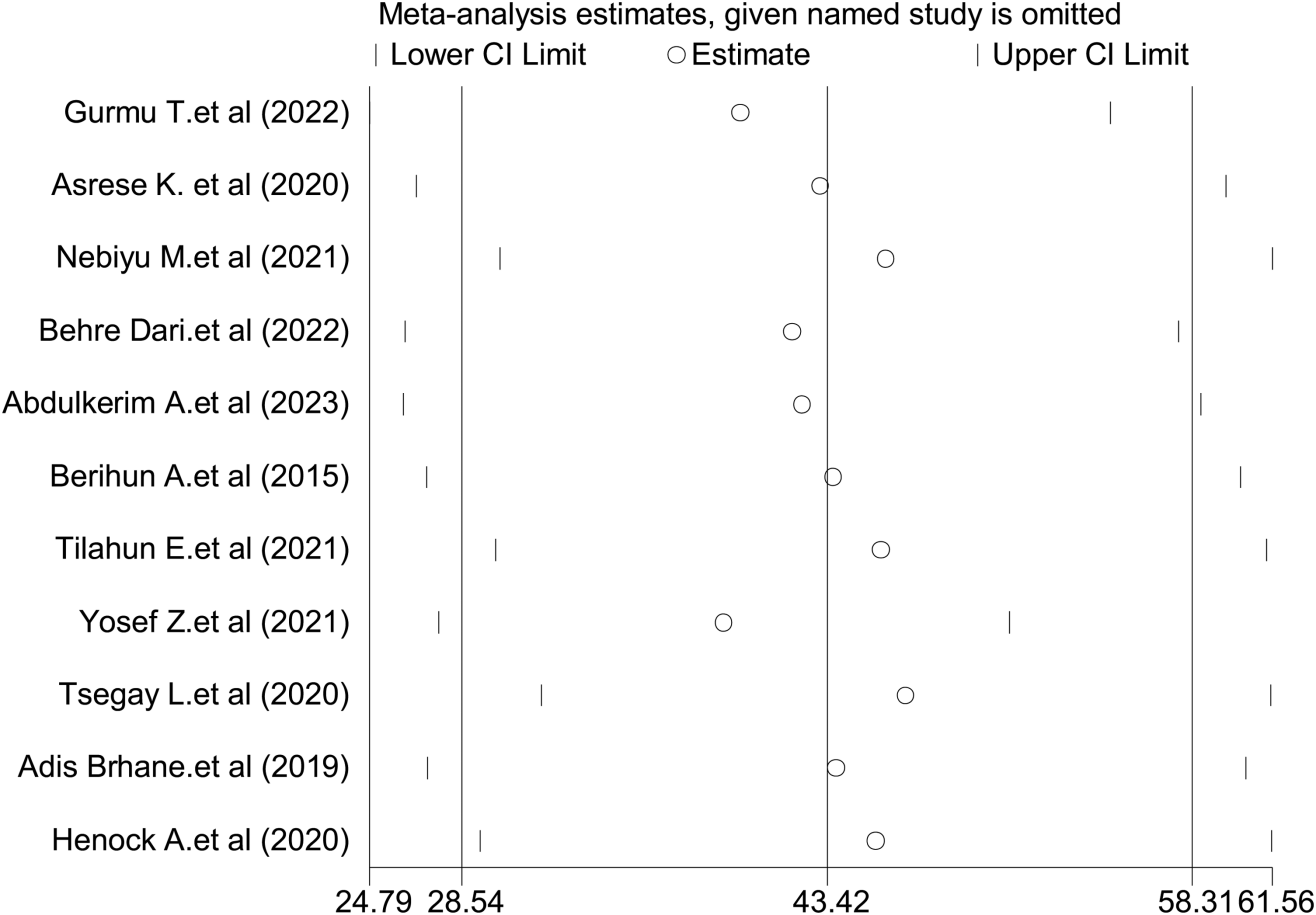
The pooled prevalence of internet addiction among university students in Ethiopia when one study omitted from the analysis a step at a time.

Leave-one-out sensitivity analysis was performed to address the possible impact of any particular study on the overall pooled effect. There was no significant influence of any specific study on the overall prevalence of IA among University students in Ethiopia (43.42%) (Figure 3).

### 3.6 Meta-regression analysis

The causes of the heterogeneity could lie in the systematic differences between the included studies in terms of measurement instruments and inclusion/exclusion criteria. Meta-regression analysis was performed for studies separately based on region, sample size, and sampling techniques (method). The results showed that the region, sample size, and sampling techniques (method) did not contribute to the heterogeneity between studies (Table 3).

**Table 3:** Meta-regression analysis of factors affecting between-study heterogeneity.

Heterogeneity sources	Coefficients	Standard error	*P*-value
Region	1.320211	0.8439949	0.674
Sample size	1.000313	0.0026112	0.907
Sampling techniques (method)	1.47238	1.764661	0.754

### 3.7 Publication bias

**Figure 4:**
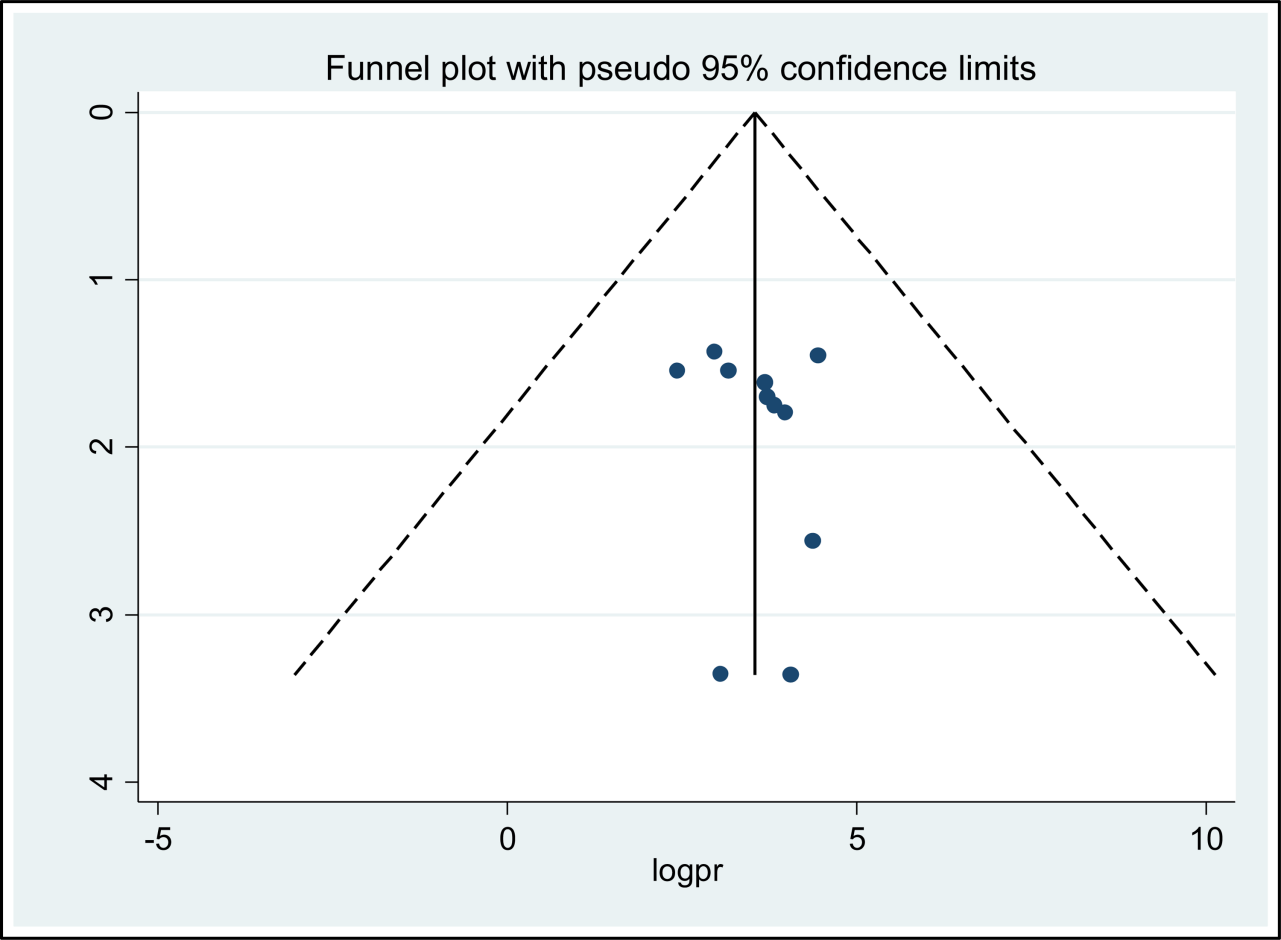
Forest plot displaying the asymmetrical distribution of the included studies.

The presence of publication bias was evaluated through both visual and objective methods. Visually, the funnel plot displayed an asymmetrical distribution of the included studies, indicating the potential presence of publication bias. To further investigate this, the Eggers regression test (*P* = 0.56) and the Beggs rank correlation test (*P* = 0.12) were employed as objective measures. The results of these tests did not provide any evidence to support the presence of publication bias (Figure 4).

### 3.8 Internet addiction and factors associated

**Figure 5:**
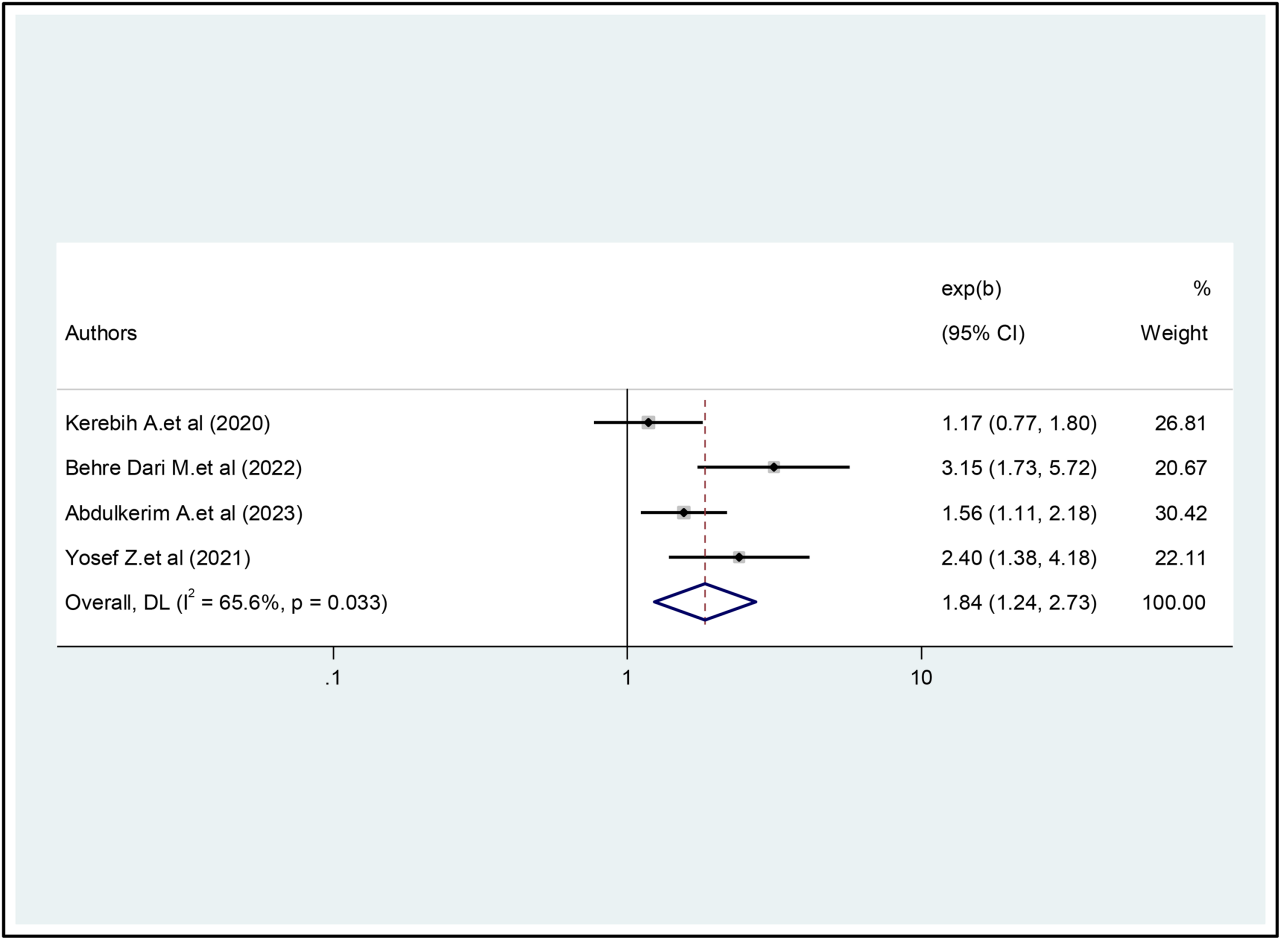
The pooled association between playing online games and internet addiction.

In this study, a total of 11 studies (18–28) were included in the analysis of Internet addiction and its associated factors. Three factors were evaluated for meta-analysis and high heterogeneity was observed in online gaming use, depression, and current khat chewing factors. In a meta-analysis of four studies (19, 21, 22, 25), online game use was found to be significantly associated with Internet addiction. Students who played mobile games online were 1.84 times more likely to develop Internet addiction than their peers or those who did not play mobile games (OR = 1.84, 95% CI = 1.24, 2.73). There was moderate heterogeneity between studies in the random effects model (I^2^ = 65.6%, *P* = 0.033) (Figure 5).

**Figure 6:**
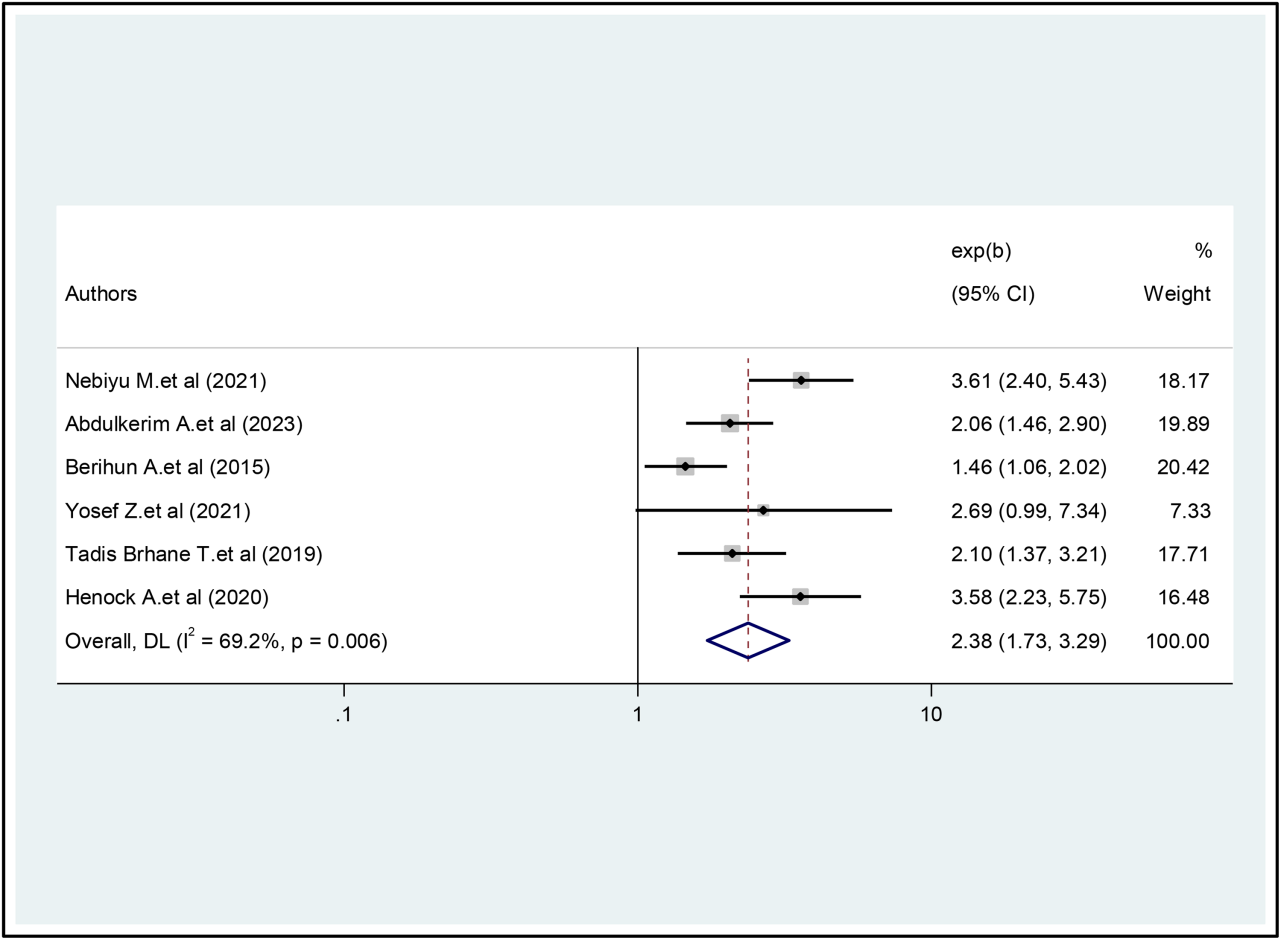
The pooled association between depression and internet addiction.

To estimate the association between depression and Internet addiction, six studies were included (20, 22, 23, 25, 27, 28). According to this meta-analysis, depression was significantly associated with Internet addiction. Study participants with depression were 2.38 times more likely to develop Internet addiction than participants without depression (OR = 2.38, 95% CI = 1.73, 3.29). There was moderate heterogeneity between studies in the random effects model (I2 = 69.2%, *P* = 0.006) (Figure 6).

**Figure 7:**
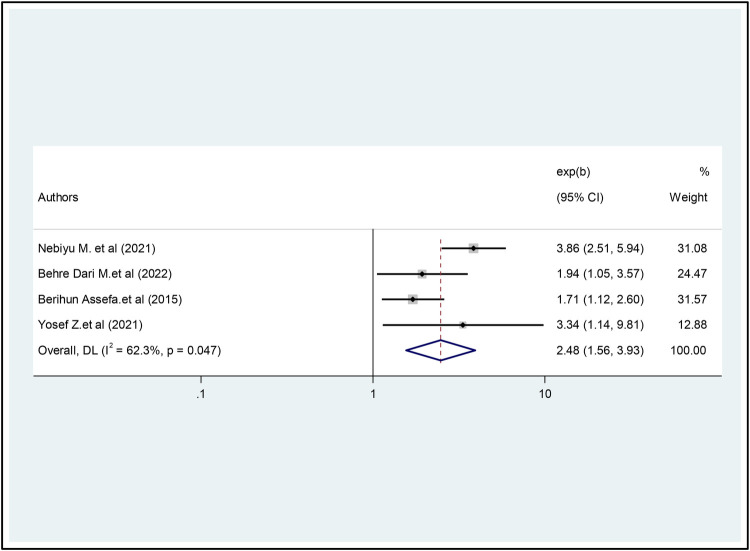
The pooled association between current khat chewing and internet addiction.

Similarly, four studies were included (20, 21, 23, 25) to estimate the association between IA and the current khat chewing status of students. The results of this four-article meta-analysis examined the association between current khat chewing status and internet addiction. According to this meta-analysis, current khat chewing was significantly associated with IA. Those who chewed khat currently had higher odds of having Internet Addiction than those who were not (OR = 2.48, 95% CI = 1.56, 3.93) (Figure 7).

## 4 Discussions

This meta-analysis study aimed to examine the prevalence of Internet addiction and its associated factors among university students in Ethiopia. A total of 11 studies were included in the review. The results of this study showed that the overall pooled prevalence of Internet addiction among Ethiopian university students was 43.42% (95% CI = 28.54, 58.31). In the final model, depression and engagement in online gaming were identified as significant factors associated with Internet addiction. Additionally, current khat chewing was found to be an independent predictor of Internet addiction. The prevalence of Internet addiction observed in this study was consistent with the prevalence rates reported in India at 42.9% (29), and Jordan at 40% (30).

The current study found a higher prevalence of Internet addiction compared to previous studies conducted in the prevalence Gulf States at 33% (31), the prevalence in Iran at 31.5% (32), the prevalence in China at 11.3% (33), the prevalence in Southeast Asia at 20% (34), and carried out in the healthcare sector were working people at 9.7% prevalence (35). Furthermore, the prevalence of Internet addiction was found to be higher in this study than another meta-analysis in Japan at 38.2% prevalence (36) and Greece at 12% prevalence (37).

The observed differences could potentially due to differences in socio-demographic characteristics or different approaches to assessing Internet addiction. These variations may include factors such as the use of the Internet Addiction Test (IAT), discrepancies in assessment tools, differences in mental health policies, and differences in time intervals between studies.

When analyzing the subgroups by region, a higher prevalence of the IA was observed in the Amhara region, followed by Oromia, while a lower prevalence was observed in Addis A. In addition, a subgroup analysis was performed based on the region sampling methods used. The prevalence was found to be relatively higher in studies that used probability sampling methods than in studies that used non-probability sampling methods. In this particular study, it was found that students who engaged in online gaming were 1.84 times more likely to develop internet addiction compared to their peers. This finding is consistent with similar studies conducted on college students in Taiwan and at three medical schools in three different countries, namely Croatia, India, and Nigeria (10, 38). Similar results have also been reported in Greek University studies and other research studies (13, 37).

Similarly, the study's results suggest that students diagnosed with depression are 2.38 times more likely to develop internet addiction than their non-depressed peers. Previous research in this area has consistently shown that individuals with depression are more susceptible to Internet addiction than individuals without psychological distress (30, 36, 39). This phenomenon may be explained by Khantzian's self-medication hypothesis (40), which posits that university students suffering from depression may use the Internet as a coping mechanism for their psychological distress. As a result, they may increasingly increase their internet usage, ultimately leading to addiction. Additionally, the study shows that university students who currently chew khat are 2.48 times more likely to develop internet addiction than their peers who do not chew khat. This result is consistent with previous research conducted among Greek university students (13), and in Japan (36).

## 5 Strength and limitations

To the best of our knowledge, this study is the first meta-analysis to examine the combined prevalence of Internet addiction (IA) among students enrolled in Ethiopian universities. The majority of studies included in this meta-analysis were considered to be of moderate quality, and due to their diverse geographical location in Ethiopia, the sample population is representative of Ethiopian university students. All searches used standardized tests and identical cutoff values. However, it is important to acknowledge certain limitations. While Internet addiction encompasses a wide range of online activities, this review uses the term IA as a general description. Furthermore, the literature on IA uses various terminologies to clarify this phenomenon.

## 6 Conclusions

The results of this review indicate that Internet addiction (IA) is a significant public health problem in Ethiopia. Approximately one-third of university students in Ethiopia are affected by IA. Factors associated with IA include depression, online gaming, and current khat use. To address this issue, public awareness campaigns can be carried out to educate the public about the seriousness and negative consequences of impact assessment. The aim of these campaigns should be to inform adults about the phenomenon of IA, its possible dangers, and its symptoms. Furthermore, all stakeholders need to collaborate and develop effective, adaptive, ethical, and sustainable interventions to combat IA.

## Data Availability

The dataset and all the relevant files are found by the primary author and can be gained from the authors upon convincing request.

## Data availability statement

The original contributions presented in the study are included in the article/Supplementary Material, further inquiries can be directed to the corresponding author.
