# Supplementary material for: Prevalence of internet addiction and associated factors among university students in Ethiopia: systematic review and meta-analysis
Source: Front Digit Health. 2024 Sep 11;6:1373735. doi: 10.3389/fdgth.2024.1373735 (PMC11422350; doi:10.3389/fdgth.2024.1373735)
Supplement: Supplementary file 2 [file Table2.docx]

**Supplementary 2**. Quality assessment for the included Studies

| **Item** | **Clearly defined inclusion** | **Describe the study setting and participant** | **Valid and reliable exposure measurement** | **Objective and standard criteria for measurement** | **Identified confounder** | **Strategies to deal with confounders** | **Valid and reliable outcome measurement** | **Appropriate static analysis** | **No of ‘yes’ ‘** |
| --- | --- | --- | --- | --- | --- | --- | --- | --- | --- |
| Gurmu T. et al | Yes | Yes | No | Yes | Yes | No | Yes | Yes | 6/8=75 |
| Asrese K. et al | Yes | Yes | Yes | Yes | No | No | Yes | Yes | 6/8=75 |
| Nebiyu M.et al | Yes | Yes | No | Yes | Yes | No | Yes | Yes | 6/8=75 |
| Behre Dari. et al. | Yes | Yes | No | Yes | Yes | Yes | Yes | Yes | 7/8=87.5 |
| Abdulkerim A. et | Yes | Yes | No | Yes | Yes | Yes | Yes | Yes | 7/8=87.5 |
| Berihun A.et al | Yes | Yes | Yes | Yes | Yes | No | Yes | Yes | 7/8=87.5 |
| Tilahun E.et al | Yes | Yes | No | Yes | Yes | Yes | Yes | Yes | 7/8=87.5 |
| Yosef Z.et al | Yes | Yes | Yes | Yes | No | No | Yes | Yes | 6/8=75 |
| Tsegay L.et al | Yes | Yes | No | Yes | Yes | Yes | Yes | No | 6/8=75 |
| Adis Brhane. et al. | Yes | Yes | Yes | Yes | No | Yes | Yes | No | 6/8=75 |
| Henock A.et al. | Yes | Yes | Yes | Yes | No | No | Yes | Yes | 6/8=75 |
