# Supplementary material for: Prevalence of internet addiction and associated factors among university students in Ethiopia: systematic review and meta-analysis
Source: Front Digit Health. 2024 Sep 11;6:1373735. doi: 10.3389/fdgth.2024.1373735 (PMC11422350; doi:10.3389/fdgth.2024.1373735)
Supplement: Supplementary file 3 [file Table3.docx]

**Supplementary 3**. Risk of bias assessment for the included studies

| **Item** | **External validity** | | | | **Internal validity** | | | | | |  | |
| --- | --- | --- | --- | --- | --- | --- | --- | --- | --- | --- | --- | --- |
|  | Representativeness of the target population | Representativeness of the sampling frame | Radom sampling or census | Minimal response bias | Data were collected directly | Acceptable case definition used in the study | Valid and reliable measurement t | The same mode of data collection for all study subject | Appropriate length of prevalence period for parameter of interest | Appropriate numerators and denominator s of interest | No of yes | Sum ery of risk of bias |
| Gurmu T. et al | Yes | Yes | Yes | Yes | No | No | Yes | Yes | Yes | Yes | 8 | Low- risk |
| Asrese K. et al | Yes | Yes | No | Yes | Yes | No | Yes | Yes | Yes | Yes | 8 | Low- risk |
| Nebiyu M.et al | Yes | Yes | No | Yes | Yes | Yes | Yes | Yes | Yes | Yes | 9 | Low – risk |
| Behre Dari. et al. | Yes | Yes | No | Yes | Yes | Yes | Yes | Yes | Yes | Yes | 9 | Low- risk |
| Abdulkerim A. et al | Yes | Yes | Yes | Yes | Yes | No | Yes | Yes | Yes | Yes | 9 | Low- risk |
| Berihun A.et al | Yes | Yes | No | Yes | Yes | Yes | Yes | Yes | Yes | Yes | 9 | Low- risk |
| Tilahun E.et al | Yes | Yes | Yes | Yes | No | No | Yes | Yes | Yes | Yes | 8 | Low- risk |
| Yosef Z.et al | Yes | Yes | No | Yes | Yes | Yes | Yes | No | Yes | Yes | 8 | Low- risk |

| Tsegay L.et al | Yes | Yes | Yes | Yes | No | Yes | Yes | No | Yes | Yes | 8 | Low- risk |
| --- | --- | --- | --- | --- | --- | --- | --- | --- | --- | --- | --- | --- |
| Adis Brhane. et al. | Yes | Yes | Yes | Yes | No | No | Yes | Yes | Yes | Yes | 8 | Low-risk |
| Henock A.et al. | Yes | yes | No | Yes | Yes | Yes | Yes | Yes | Yes | Yes | 9 | Low- risk |
